# A Rare Presentation of Pneumococcal Meningitis with Acute Bilateral Vision Loss: A Case Report

**DOI:** 10.5811/cpcem.63985

**Published:** 2026-10-13

**Authors:** Adam Anderson, Alexander Piszker, Eric Applebach, Reid Mitchell, Aji Yagou Jeng

**Affiliations:** Trinity Health West Michigan Emergency Medicine Residency, Muskegon, Michigan

**Keywords:** pneumococcal meningitis, bilateral vision loss, acute ischemic optic neuropathy, case report

## Abstract

**Introduction:**

Bacterial meningitis affects about 1.2 million people worldwide each year. *Streptococcus pneumoniae* is responsible for the majority of cases of bacterial meningitis in the United States and Europe, with visual impairment seen in 6.3% of patients. The etiology of acute vision loss in the setting of bacterial meningitis remains wide-ranging, most often occurring in the setting of endogenous endophthalmitis, optic neuropathy, and cortical blindness. Retinal necrosis is rare, and typically associated with viral meningitis, but has not been reported in bacterial meningitis.

**Case report:**

A 66-year-old man with headache, malaise, and intermittent blurry vision, initially diagnosed with viral upper respiratory infection and presumed trigeminal neuralgia, presented to the emergency department with altered mental status, meningismus, and progressive bilateral vision loss. He was diagnosed with bacterial meningitis, and subsequent cerebrospinal fluid and blood cultures were positive for *S pneumoniae*. Ophthalmologic evaluation demonstrated bilateral nonreactive pupils, retinal ischemia, optic nerve pallor, and retinal necrosis but no intraocular inflammation. Brain magnetic resonance imaging showed multifocal cortical ischemic infarcts thought to be of embolic source. His hospitalization was complicated by mitral valve regurgitation, endocarditis, and cardiac tamponade requiring extensive surgical repair.

**Conclusion:**

This case represents a rare presentation of bilateral retinal ischemia and necrosis in the setting of *S pneumoniae* meningitis without evidence of endophthalmitis. Awareness of atypical ocular presentations and differential diagnoses is vital in providing early diagnosis, treatment and specialist involvement.

## INTRODUCTION

Bacterial meningitis is a life-threatening condition affecting about 1.2 million people worldwide each year.^1^ *Streptococcus pneumoniae* is responsible for approximately 66% of all cases of bacterial meningitis in the United States and Europe.^2^ Headache is the most common symptom, present in more than 85% of patients; seizures and focal neurologic deficits occur in 25% to 30%.^1^ One systematic review and meta-analysis of bacterial meningitis complications found that visual impairment occurred in 6.3% of patients.^3^ The differential diagnosis for acute vision loss in the setting of bacterial meningitis is broad, with case reports discussing etiologies ranging from endogenous endophthalmitis to cortical blindness to acute neuritis.^4^–^6^ Acute vision loss due to bacterial meningitis most often occurs in the setting of endogenous endophthalmitis, optic neuropathy, and cortical blindness. Retinal necrosis is a rare complication, and while it can present as a component of endogenous endophthalmitis it is typically indicative of an underlying viral process. Our case highlights a unique presentation of acute bilateral visual impairment in the setting of *S pneumoniae* bacterial meningitis, with findings of retinal ischemia and necrosis.

## CASE REPORT

A 66-year-old retired, active man with no pertinent past medical history initially presented to urgent care with five days of upper respiratory symptoms, headache, and intermittent blurry vision. Tests for COVID-19 and influenza were negative. He was diagnosed with viral upper respiratory tract infection, prescribed fluticasone nasal spray, and discharged with instructions for routine primary care follow-up. Four days later, he presented to the emergency department (ED) for headache, jaw pain, and generalized malaise. He was afebrile with normal heart rate and blood pressure. On physical exam, he was well appearing without meningismus or other focal neurologic deficits. Computed tomography of the head was negative, erythrocyte sedimentation rate was mildly elevated at 28 mm/hour (reference range, 0–20 mm/hour), and white blood cell (WBC) count was elevated at 15.8 K/mcL (4.0–10.0 K/mcL). He was diagnosed with suspected trigeminal neuralgia and discharged on prednisone and carbamazepine.

Two days later, he returned to the ED with headache, weakness, altered mental status, and bilateral vision loss. His vitals showed the following: temperature, 37.4 °C; heart rate, 107 beats per minute; and blood pressure, 105/72 mm Hg. He was confused with orientation to name only and had nuchal rigidity and a presumed new heart murmur. Ocular evaluation demonstrated bilateral dilated, nonreactive pupils with severely diminished visual acuity (light perception only). Lumbar puncture with cerebrospinal fluid (CSF) studies revealed elevated protein at 173 mg/dL (reference range, 12–60 mg/dL), normal glucose at 64 mg/dL (40–70 mg/dL), and an elevated WBC count of 1,306 mm^3^ (0–5 mm^3^) with neutrophilic predominance. Gram stain showed moderate Gram-positive cocci similar to pneumococci. Serum WBC count was elevated at 23.5 K/mcL (reference range, 4.0–10.0 K/mcL). The patient received a 30 mL/kg bolus of lactated Ringer’s solution, ceftriaxone, vancomycin, acyclovir, and dexamethasone, and he was admitted to the intensive care unit for empiric treatment of bacterial meningitis.

During admission, ophthalmology was consulted. Their examination documented ongoing bilateral vision loss with 5 mm, minimally reactive pupils. Dilated fundoscopic exam revealed severe central retinal ischemia with scattered hemorrhages and complete pallor of bilateral optic nerves and maculae, suggestive of severe, bilateral ischemic optic neuropathy. Although acute retinal necrosis was initially thought to be related to meningitis, clinicians considered a concomitant giant cell arteritis. Subsequent evaluation by a retinal specialist revealed acute retinal necrosis, which was likely caused by varicella-zoster virus (VZV) or herpes simplex virus (HSV). Neither the ophthalmologist, nor the retinal specialist, found evidence of cell or flare in the anterior or posterior chambers. Additionally, polymerase chain reaction testing of CSF for VZV and HSV were negative. Vitreous culture was negative after five days and sample was insufficient for further polymerase chain reaction **(**PCR) testing.

*CPC-EM Capsule*

What do we already know about this clinical entity?

*Visual loss is a rare complication of pneumococcal meningitis, usually attributed to endophthalmitis, optic neuropathy, or cortical blindness.*

What makes this presentation of disease reportable?

*This is the first reported case of bilateral retinal ischemia and necrosis in the setting of pneumococcal meningitis without evidence of endophthalmitis.*

What is the major learning point?

*Pneumococcal meningitis can cause retinal ischemia and necrosis, even without endophthalmitis.*

How might this improve emergency medicine practice?

*Recognizing atypical ocular complications of bacterial meningitis can help guide timely diagnosis and treatment.*

Encephalitis panel, CSF, and blood cultures returned positive for *S pneumoniae.* Antibiotic therapy was guided by infectious diseases consult with recommendations to continue vancomycin and ceftriaxone. Acyclovir was discontinued. Vancomycin was discontinued after three days as the cultures were adequately sensitive to ceftriaxone.

On hospital day 3, brain magnetic resonance imaging (MRI) revealed a focus of acute ischemia in the left subcortical occipital lobe and an oblong focus of acute ischemia in the right posterior cerebellum, likely secondary to an embolic phenomenon. Neurology was consulted for stroke and possibility of giant cell arteritis, with recommendations for dual antiplatelet therapy for 21 days followed by aspirin monotherapy, as well as a temporal artery biopsy. The general surgery team did not pursue the biopsy because they considered it to be unlikely to change diagnosis or therapy. On hospital day 4, an echocardiogram revealed findings of aortic stenosis, posterior mitral valve regurgitation, and mitral valve vegetation, consistent with endocarditis. Cardiothoracic surgery consult did not recommend surgical intervention given the patient’s unresolved neurologic status.

On hospital day 7, the patient developed chest pain with troponin elevation of 150 ng/L (reference range, < 21 ng/L) with no ischemic electrocardiographic changes. Vitals signs were within normal limits. Computed tomography angiography of the chest showed pericardial effusion with concern for cardiac tamponade. An emergency repeat echocardiogram showed right ventricular dynamic collapse, confirming cardiac tamponade. He underwent emergent pericardiocentesis with removal of 400 mL of blood, resolving the tamponade. The cardiothoracic surgery team concluded that the tamponade developed secondary to mitral valve endocarditis eroding through the aortomitral curtain into a 5.8-cm ascending aortic aneurysm. Specialists at tertiary centers concluded that the patient’s disease progression represented a terminal condition given the required extensive surgical interventions. The patient chose to pursue comfort measures. Antibiotics were discontinued, and he was discharged home on hospice.

While in hospice, the patient exhibited unexpected improvement in his clinical condition, leading him to seek a second opinion regarding cardiothoracic surgical options. Two weeks after initial hospital discharge, he underwent aortic and mitral valve replacement, two-vessel coronary artery bypass grafting, ascending aorta replacement, and pericardial patch repair of proximal aortic abscess/fistula. He had a remarkably uneventful postoperative recovery. To date, he is stable and recovering at home but remains blind with no light perception.

## DISCUSSION

The incidence of pneumococcal meningitis in the United States is 0.3 per 100,000 people, with reported mortality rate as high as 37%.^2^,^7^ For those who survive, devastating neurologic complications can occur in up to 52% of patients.^2^ In the rare case of concomitant acute vision loss, establishing a differential diagnosis provides an opportunity to review the potential causes of vision loss and identify which features of each pathologic process correlate with the patient’s presentation.

There was much discussion about the underlying etiology of our patient’s bilateral visual impairment. The retinal specialist considered the patient’s ocular findings to be consistent with acute retinal necrosis, implying concomitant viral infection in addition to the patient’s bacterial meningitis. Acute retinal necrosis is caused by VZV and HSV, characterized by retinal inflammation, uveitis, vasculitis, and necrosis.^8^ Fundoscopic examination typically demonstrates sharply demarcated areas of necrosis with evidence of inflammation of the anterior and posterior chambers.^8^ Treatment consists of systemic antivirals and, occasionally, intravitreal antivirals.^8^ Involvement of both eyes is not uncommon and can lead to permanent bilateral vision loss.^8^ This can also be seen in the setting of viral meningitis but has not been reported as a complication of bacterial meningitis.^9^ Contrary to ophthalmologist opinion, the infectious diseases specialist maintained that a concomitant bilateral viral retinal process in the setting of a severe bacterial infection would be unlikely. Additionally, CSF PCR was negative for HSV and VZV, and no inflammation was observed in either the anterior or posterior chambers, further supporting an unlikely diagnosis of acute retinal necrosis. The infectious diseases specialist concluded that the overwhelming burden of the systemic bacterial infection directly led to retinal injury and necrosis.

Another important consideration provided by neurology was the possibility of acute cortical blindness, an uncommon but recognized complication of bacterial meningitis.^10^ Inflammation and edema are involved in the disease process, and vasculitis leads to cortical injury with focal necrosis.^11^ Given that this is a cortical lesion, pupillary light reflexes and extraocular movements typically remain intact, and fundoscopic evaluation remains normal.^12^ Although our patient did have evidence of cortical ischemia on MRI, it was likely secondary to septic embolic phenomena, and his loss of pupillary reflexes; therefore, his retinal findings made cortical blindness unlikely to be the primary cause of his vision loss.

Given the patient’s presentation with headache and jaw pain, an important differential was acute ischemic optic neuropathy (AION), which can present as either arteritic or nonarteritic. Nonarteritic anterior ischemic optic neuropathy, which is typically idiopathic, accounts for 90% to 95% of cases of AION; investigators hypothesize that circulatory insufficiency to the optic disc ultimately leads to ischemia and painless vision loss.^8^ The vision loss is typically mild, with the progressive form worsening over weeks.^8^ Arteritic anterior ischemic optic neuropathy is a manifestation of giant cell arteritis, leading to disc ischemia and retinal necrosis, causing severe vision loss, which is typically unilateral but sometimes bilateral and sudden.^8^,^13^ This was deemed unlikely as our patient had an erythrocyte sedimentation rate < 50 mm/hour, which did not rule out giant cell arteritis but made it far less likely.^14^

An uncommon but important diagnostic consideration was central retinal artery obstruction (CRAO). This ocular emergency typically causes painless, unilateral vision loss (98% to 99% of cases).^8^ Examination shows a central area of pallor with a persistent cherry-red spot overlying the macula due to its thinner nerve layer.^8^ This appearance persists for six weeks, after which the retinal pallor spontaneously resolves without progressing to necrosis.^8^ Although our patient was at increased risk for embolic phenomena, his retinal findings were not consistent with CRAO.

## CONCLUSION

Ultimately, the etiology of our patient’s ocular symptoms and findings remain unclear. To our knowledge, this is the only reported case of bacterial meningitis-associated retinal necrosis without evidence of endophthalmitis. Due to the severity of disease burden, we suspect a pathophysiologic process with both localized and cortical insults. We postulate that the severe systemic and localized inflammation from pneumococcal meningitis led to vasculitis and retinal necrosis. Although most of the diagnoses considered in this case are beyond the scope of the emergency clinician, general knowledge of their occurrence and basic pathophysiology can be vital in providing early diagnosis, treatment, and specialist involvement. Such early interventions are imperative both to guide initial diagnosis and treatment and preserve vision while decreasing overall morbidity and mortality.
